# Immunotherapy for penile cancer

**DOI:** 10.4155/fsoa-2017-0031

**Published:** 2017-05-02

**Authors:** Carlo Buonerba, Martina Pagliuca, Francesca Maria Vitrone, Ilaria Ascione, Ivana Elefante, Vittorio Riccio, Francesca Romano, Sabino De Placido, Giuseppe Di Lorenzo

**Affiliations:** 1 Dipartimento di Medicina Clinica e Chirurgia, Università Federico II di Napoli, Naples, Italy; 2 Istituto Zooprofilattico Sperimentale del Mezzogiorno, Portici, Italy

**Keywords:** environment, HPV, immunotherapy, nivolumab, penile cancer

## Background

Penile carcinoma is a rare disease, with incidence rates varying in the range of 1–10 cases per 100,000 men depending on ethnicity, geographic area, cultural background and social habits [1,2]. Tumorigenesis of penile carcinoma is governed by a complex interplay of multiple causative factors. These include initiating agents, such as tobacco toxins, UV radiation and, possibly, household contaminants from solid fuel combustion, which have been implicated in carcinoma of the cervix [3], as well as promoting agents, such as cytokines related to chronic inflammation and high-risk HPV, mainly HPV-16 and HPV-18 [2], which are well known for their major etiopathogenetic role in cervix carcinomas [4]. In patients with carcinoma of the penis, keratinizing squamous cell and verrucous lesions harbor high-risk HPV only in 30% of cases and coexist with squamous cell hyperplasia and/or lichen sclerosus, while basaloid and warty carcinomas, which are composed of small, undifferentiated basaloid cells with koilocytic changes, harbor HPV in 80–100% of cases [2]. Positivity to high-risk HPV has both prognostic and biological implications in penile cancer. In fact, HPV infection may be associated with better outcomes in penile cancer men, as reported in a retrospective study of 171 patients showing a 5-year cancer-specific survival rate of 78 and 93%, respectively, in the high-risk HPV-negative subgroup versus the high-risk HPV-positive subgroup (log rank test p = 0.03) [5]. Furthermore, while HPV-positive tumors express more frequently HER3 and cytoplasmic Akt1, HPV-negative tumors express more frequently phosphorylated EGFR [6], which is consistent with the negative prognostic effect associated with presence of phosphorylated EGFR [7].

## The role of HPV as a potential target for immunotherapy

The HPV proteins E6 and E7 play a key role in HPV-mediated carcinogenesis. In addition to inactivating p53, E6 can bind to transcription factors (myc), autocrine motility factors that regulate cell adhesion and polarity (paxillin), apoptosis-inducing factors (Bcl2) and replication and DNA repair factors (mcm7), while the E7 protein inactivates the retinoblastoma tumor-suppressor protein via proteasome-dependent degradation and causes p16INK4a overexpression, which can be detected on immunohistochemistry and can be employed as a reliable diagnostic marker of high-risk HPV infection [8]. Interestingly, the p16INK4a protein is overexpressed both in intraepithelial and invasive lesions [9,10], and can serve as a reliable diagnostic histologic biomarker of HPV infection in penile cancers. On the basis of the established etiopathogenetic role of HPV in a subgroup of penile cancer patients, we wish to speculate here that HPV-associated antigens have the potential to provide specific targets for an immunotherapy approach in men with penile cancer. At the present time, two vaccines based on HPV L1 virus-like particles are commercially available and approved in young women in order to prevent HPV infection, that is Gardasil ^®^(Merck & Co., NJ, USA) and Cervarix^®^ (GlaxoSmithKline, England, UK). While Gardasil contains virus-like particles from HPV-16 and HPV-18, but also from low-risk carcinogenic genotypes 6 and 11, which cause benign genital warts, Cervarix contains virus-like particles from HPV-16 and HPV-18 only [11]. Spontaneous clearance of high-risk HPV occurs in approximately a third of women after 6 months and in approximately half of the women after 12 months [11]. Although available preventive anti-HPV vaccines are able to induce both antibody and cellular responses, they are not able to improve spontaneous HPV clearance rate [11], so they cannot be considered as candidates for an immunotherapy approach in HPV-mediated tumors. In fact, while HPV L1 protein is predominantly expressed in terminally differentiated keratinocytes, expression of the E6 and E7 proteins is retained through all of the epithelial layers and through multiple stages of infection. As a result, an immune response against E6 and E7 antigens may be effective to clear E6- and E7-expressing neoplastic cells [12].

## Future perspective: VGX-3100 & anti-PD1/PD-L1 agents

The novel immunotherapy agent VGX-3100 (Inovio Pharmaceuticals, PA, USA), which is delivered via electroporation, is based on two property DNA synthetic plasmids that encode the E6 and E7 genes of HPV-16 and HPV-18 [12]. Electroporation uses brief electric pulses to cause transient and reversible permeabilization of the cell membrane, which optimizes transfection of nucleic acids, with a 100–1000-fold enhancement of plasmid delivery and gene expression [12]. VGX-3100 was tested in a pivotal Phase I study in 18 women with recurrent cervical intraepithelial neoplasia (CIN) grade 2 or 3, showing encouraging results in terms of HPV-specific CD8^(+)^ and CD4^+^ T-cell response [12]. In a subsequent double-blind, placebo-controlled Phase IIb study [13], 167 patients with CIN2/3 associated with HPV-16 and HPV-18 were randomly assigned in a 3:1 ratio to receive 6 mg VGX-3100 (n = 125) or 1 ml placebo solution (n = 42), both given intramuscularly at 0, 4 and 12 weeks. The primary objective of the study, that was improvement of histopathological regression rate of CIN 2/3 lesions, was met in the modified intention-to-treat analysis, with 55 (48·2%) of the 114 patients receiving VGX-3100 and 12 (30·0%) of the 40 placebo recipients showing regression to CIN 1 or no disease. The safety profile of VGX-3100 was excellent, with the majority of patients showing injection-site reactions, and erythema being significantly more frequent in the VGX-3100 group (98/125, 78·4%) with respect to the placebo group (24/42, 57·1%; p = 0·007). While VGX-3100 may be useful to avoid morbidity of surgical treatment in women with CIN2/3 cancers, this agent may provide survival benefits in patients with limited therapeutic options such as those with penile carcinoma. As we reported previously, prognosis of penile cancer is excellent in patients with noninvasive disease, while in patients with invasive tumors 5-year cancer-specific survival rates vary in the ranges of 75–93%, 40–70%, 33–50% and 20–34% in men with cN0, cN1, cN2 and cN3 disease [14]. Prognosis of patients requiring systemic chemotherapy for advanced disease is poor – approximately 6–12 months [15,16]. We speculate that a potential setting of experimental use of VGX 3100 in a clinical trial may include men with p16INK4a-positive penile cancer who have undergone complete surgical resection, but are at significant risk of disease recurrence. Conversely, we speculate that in men with metastatic penile cancer that tested positive for HPV 16/18, given the high burden of the disease, combination of an active, antigen-specific immunotherapy treatment such as VGX 3100 with an anti-PD (Programmed Death)-1/PD-L1 (Programmed Death-Ligand 1) agent may be beneficial. In fact, in a recently published retrospective study, 23 (62.2%) of 37 primary tumors were positive for PD-L1 expression, with a strong positive correlation of PD-L1 expression in primary and metastatic samples [17]. Of note, anti PD-1 agent nivolumab has shown efficacy in head and neck cancers, which share histologic (squamous histology) and pathogenic (HPV infection) characteristics with penile cancer [18].

In conclusion, although the industry may show little interest in rare diseases such as penile cancer, a continued effort should be made by independent investigators to contribute to advances in the treatment of such a devastating disease, given its high morbidity and mortality.
